# Multiple Sclerosis and the LIF/IL-6 Axis: Use of Nanotechnology to Harness the Tolerogenic and Reparative Properties of LIF

**DOI:** 10.5772/60622

**Published:** 2015-01-01

**Authors:** Su M. Metcalfe, Terry B. Strom, Anna Williams, Tarek M. Fahmy

**Affiliations:** 1 Cambridge University Hospitals NHS Foundation Trust, Addenbrookes Hospital, Cambridge Biomedical Campus, Cambridge, UK; 2 The Transplant Institute, Beth Israel Deaconess Medical Center, Center for Life Science (CLS), East Campus, Boston, MA, USA; 3 Centre for Regenerative Medicine, University of Edinburgh, Edinburgh, UK; 4 Department of Biomedical Engineering, Department of Immunobiology Yale School of Engineering and Applied Science and Yale School of Medicine, New Haven, CT, USA

**Keywords:** Nanomedicine, Inflammatory immune axis, LIF-nanoparticles, Multiple sclerosis, Targeted delivery, *In vivo*

## Abstract

Leukaemia inhibitory factor (LIF) plays a critical role in “stemness” versus “differentiation”, a property that underpins the core value of LIF as a therapeutic for both the treatment of autoimmune disease and for promoting tissue repair. This value can be realized using nano-engineering technology, where a new generation of tools can, with unprecedented ability, manipulate biological functions. One striking example is the treatment of multiple sclerosis (MS). The underpinning biology is the newly identified LIF/IL-6 axis in T lymphocytes, which can tilt the behaviour between immune tolerance versus immune attack. This LIF/IL-6 axis is ideally suited to nanotherapeutic manipulation, given its inherent mechanistic simplicity of two mutually opposing feed-forward loops that determine either tolerogenic (LIF) or inflammatory (IL-6) immunity. Using LIF that is formulated in biodegradable nanoparticles (LIF-NP) and targeted to CD4+ T cells, the axis is harnessed towards immune tolerance. This has implications for the treatment of autoimmune diseases, where the clinical burden is immense. It encompasses more than 100 diseases and, in the USA alone, costs more than $100 billion in direct health care costs annually. Other properties of LIF include the promotion of healthy neuro-glial interactions within the central nervous system (CNS), where, in addition to MS, LIF-NP therapy is relevant to inflammatory neurodegenerative diseases that represent a large and increasing need within aging populations. Thirdly, LIF is a reparative growth factor that can maintain genomic plasticity. LIF-NP supports the use of stem cell-based therapies in regenerative medicine plus augment therapeutic benefits within the patient. These core properties of LIF are greatly amplified in value by the advantage of being formulated as nanoparticles, namely (i) targeted delivery, (ii) exploitation of endogenous regulatory pathways and (iii) creation of surrogate micro-stromal niches. We discuss LIF-NP as a means to harness endogenous pathways for the treatment of MS, both to reset immune self-tolerance and to promote repair of myelin that is required to support health within the nervous system.

## 1. Introduction

Nanomedicine is a new era in therapeutics, which has been enabled by fundamental advances in biology combined with progress in biocompatible nanotechnology. Nanotechnology provides access to three highly desirable therapeutic aims, namely (i) *targeted delivery of drugs or biologics*, for example of cytotoxins to cancer cells, where relatively high doses of the drug can be focused upon the tumour with reduced off-target side effects; (ii) *exploitation of endogenous regulatory systems*, such as harnessing the adaptive immune response towards immune self-tolerance in the treatment of autoimmune disease or to prevent graft rejection, or alternatively to prime aggressive immunity towards cancer cells; (iii) *creation of an artificial transient microstroma* as a supportive niche for endogenous repair, applicable following trauma or in age-related degenerative diseases, or as a supportive matrix for cells used in cell-based therapy. We consider the value of the targeted delivery of a stem cell growth factor called “leukaemia inhibitory factor” (LIF) in the context of LIF's biologic properties which are directly relevant to the treatment of multiple sclerosis (MS).

### 1.1 Why Choose LIF as a Therapeutic?

Why LIF? Firstly, LIF is a multi-functional cytokine that is involved in regulating the plasticity of a genome [for example, 1, 2]. Such plasticity plays a central role throughout life because it permits the progressive stages of lineage development within the different biological systems of an organism. Plasticity is linked to micro-environmental cues during a cell's transit through fate decision forks, for example during haematopoiesis, to provide the different cell types that make up a healthy haematopoietic system [3]. LIF's role in regulating genomic plasticity is not only central for development, but also for the recruitment of endogenous stem cells and precursor cells during the maintenance, repair and remodelling of mature tissues. Examples include bone remodelling in response to stress, and the increase in muscle mass in response to work load. The plasticity of a genome supported by LIF is linked to “pioneer factors”. These are transcription factors able to access specific genes within compacted DNA in response to growth factor signalling. Notably, the loss of the LIF receptor, gp190, is early embryonic lethal. Indeed, LIF signalling is critically involved in the full reprogramming control of biological systems, which is achieved by only a few key control factors that impact a small number of nodes (five or less) [4]. Overall, LIF is highly attractive as a therapeutic, with special relevance to the regenerative medicine arena, where harnessing of LIF using nanotechnology will progress the move towards cell-free therapeutics.

As a therapeutic, the second attraction of LIF is its role in the adaptive immune system. Here, T lymphocytes, each expressing a unique antigen-specific receptor, provide the full repertoire of antigen recognition of an individual plus the exquisite specificity of the adaptive immune response. Failsafe mechanisms ensure that the power of an immune attack is restricted to invading pathogens. Should these mechanisms malfunction, then an immune attack against the body's own tissues will lead to autoimmune disease and possibly even death. An autoimmune *protective* role for LIF has recently been discovered [5–8]. Here, LIF supports self-tolerance by supporting self-reactive tolerogenic regulatory T lymphocytes (Treg). Since an activated Treg releases more LIF, self-sustaining populations of self-reactive Treg are perpetuated.

### 1.2 The LIF / IL-6 axis – A Critical Node in Immunity that Defied Dogma

In the adaptive immune system, the finding that LIF, in addition to promoting Treg and self-tolerance, *also* directly opposes interleukin-6 (IL-6) [8] is of profound importance. IL-6 induces inflammatory immunity including immunity driven by TH17 cells which, when inappropriately activated, are strongly linked to autoimmune disease. To be able to harness the LIF / IL-6 axis in order to reset autoimmune self-tolerance would achieve an ultimate goal in autoimmune therapies.

**Figure 1.**
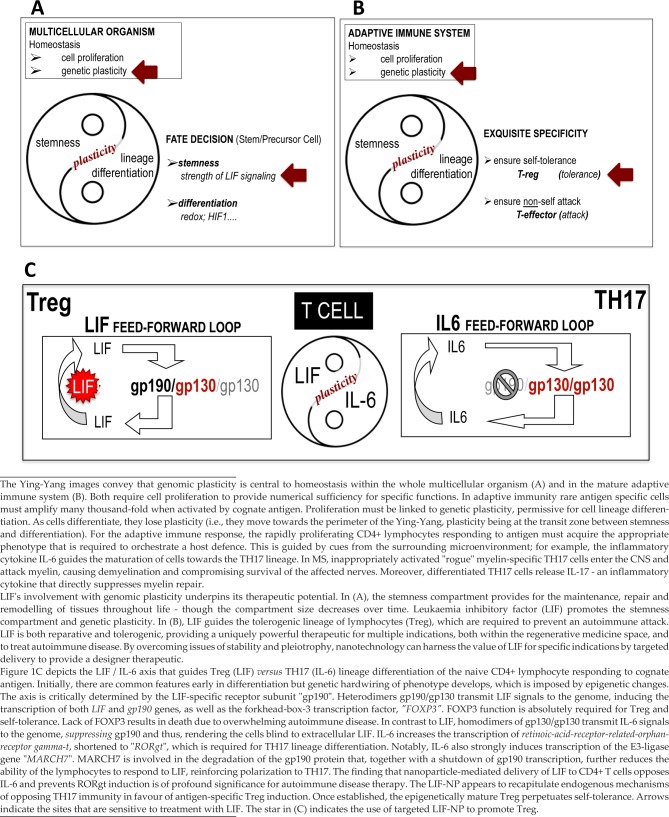
Some Biological Sites Available to Manipulation by LIF Therapy

Dogma blocked initial attempts to publish the LIF / IL-6 axis in Treg / TH17 immunity. Flawed reasoning argued that, since LIF belongs to the IL-6 cytokine family, then LIF must activate the same pathways and genes as IL-6. Biased judgement obscured the discovery that *opposing feed-forward loops of gene expression are activated by LIF versus IL-6*. The expression of gp190, the LIF-specific sub-unit of the LIF receptor, provides a pivotal mechanism for the axis (Figure 1C) [7, 8]. It is relevant to note that transforming growth factor beta (TGFβ) is required for T cell activation. This raises the question, how does TGFβ fit with the LIF / IL-6 axis? In experiments conducted by Gao et al., although activated TGFβ was required for both Treg and TH17 lineage maturation, it was LIF, *versus* IL-6, that imposed the mutually exclusive lineage-specificity [8]. Thus, TGFβ acts in combination with LIF, or IL-6, but does not specify lineage.

The field is young. Do other systems also operate under a LIF / IL-6 axis for cell fate in development, for example, during endogenous repair? If yes, then the combined properties of LIF (reparative and tolerogenic) might cooperate *in vivo* with Treg, playing a dual role – one immune, for self-tolerance and the second, non-immune, for repair. Moreover, given its proposed mechanistic simplicity – based on receptor competition between the two cytokines [7, 9–10] – the LIF / IL-6 axis may represent a previously unrecognized mechanism at the core of stemness. This could even extend to asymmetric division, given that NANOG enhances LIF signal transduction [2]. Although clearly speculative, these predications anticipate the targeted delivery of LIF as a powerful nanotherapeutic platform relevant to a wide range of indications.

Figure 1 depicts the position of genomic plasticity in homeostasis in development (Figure 1A) and in the immune system (Figure 1B), and at the LIF / IL-6 axis during CD4+ lymphocyte maturation (Figure 1C). Sites that are available for manipulation by LIF are indicated. These sites can be accessed using nanotechnology and we next present the nano-formulation of LIF which permits its use as a therapeutic.

## 2. Therapeutic Nanoparticles and the Adaptive Immune system

A short half-life normally attenuates cytokine activity *in vivo* [11] and this needs to be overcome when exploiting cytokines for therapy. Cytokine half-life may be prolonged by modifying the cytokine structure, for example by the attachment of fusion molecules such as polyethylene glycol (PEG) [12] or humanized antibody fragments [13], or by genetic engineering to create a glycosylated cytokine. An alternative approach is cytokine gene therapy. This uses viral constructs, including lentivectors, which actively integrate into genomic DNA without requiring cell replication and provide a stable, long-term expression of the gene. However, these approaches lack the sophisticated levels of control that are available when applying nanotechnology. Furthermore, none of them replicate the physics of delivery on a nanoscale, where potency of cargo is massively increased.

### 2.1 The LIF-PLGA Nanoparticle Prototype

**Figure 2.**
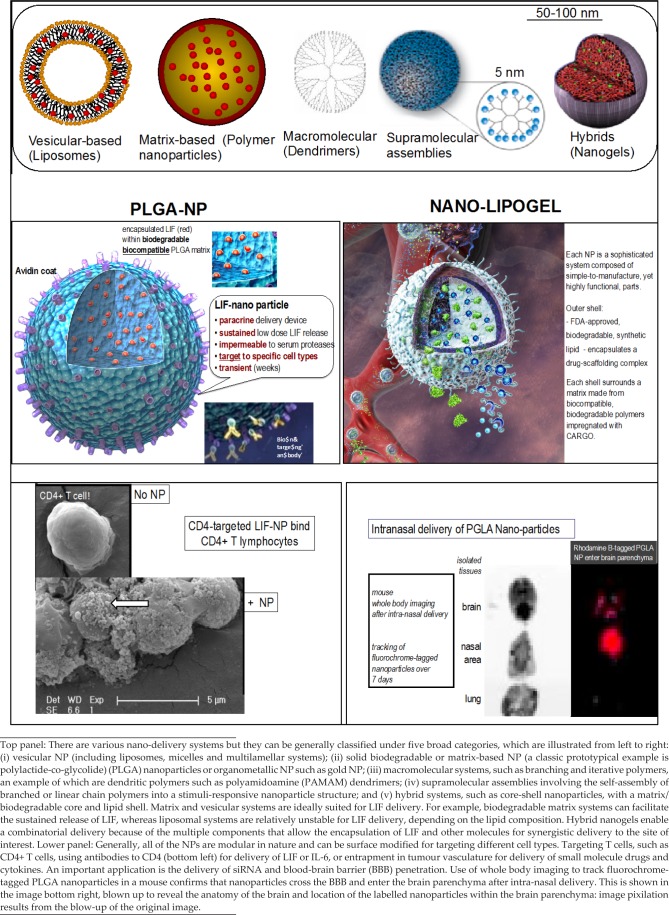
Optional Nanoparticle Formulations of LIF

The development of the LIF-PLGA nanoparticle (LIF-NP) was based on strategic reasoning. The polymer poly(lac-tide-co-glycolide) (PLGA) is already approved by the FDA for drug delivery applications due to its safety, excellent biocompatibility and “tunable” release rates. In nanoparticulate form, PLGA decorated with functional avidin groups on the nanoparticle surface, enables the modification of this surface through the robust attachment of biotinylated ligands such as PEG [14], T cell-stimulating antibodies [15], or T cell-targeting antibodies [16]. This technology is well-suited towards stimulation and manipulation of immune cell development through (i) the presence of T cell-specific cell surface molecules that can be targeted by antibodies; (ii) on the nanoparticle, presentation of multiple targeting ligands per nanoparticle, ensuring high valency and avidity of contact with targeted cellular ligands; and (iii) delivery of multiple cytokine molecules per biorecognition event to ensure a relatively high concentration of cytokine precisely within the micro-environment of the targeted cell, whilst avoiding systemic exposure to the therapeutic cytokine. Figure 2 illustrates a range of nano-structures, together with the prototypic LIF-PLGA nanoparticle.

### 2.2 Does it Work? Is Targeting Necessary? What Dose of LIF Is Delivered? In Vivo?

For the prototype, the end point of success was the demonstration of (i) target-specific delivery of cytokine; (ii) retained bioactivity specific to the released cytokine cargo; and (iii) a biological response specific to the targeted cells. All of these benchmarks were met when exploiting the LIF / IL-6 axis as a stringent model to prove bio-efficacy in a study by Park et al. [17]. When targeted to CD4+ T lymphocytes, LIF-NP, *versus* IL-6-NP, provided extracellular cues able to control T cell maturation *via* the lineage-determining master genes, *FOXP3* (Treg) and *RORγt* (TH17), respectively [18–22]. Cell targeting was necessary for the biological response and demonstration of being able to harness a fate decision in CD4+ T lymphocytes, where there is exquisite specificity for antigen combined with immune memory, led to the highly relevant question, *can LIF-NP directly oppose the IL6-driven pathway?* If yes, then the targeted delivery of LIF by LIF-NP may guide tolerogenesis – even in the face of a local inflammatory IL-6-rich micro-environment as occurs in TH17-linked autoimmune activity. The actual dose of cargo being delivered when using nanoparticles is extremely low: one milligram of PLGA nanoparticles carries around one nanogram LIF cargo. However, LIF, when formulated as LIF-NP, was >1000-fold more potent than soluble LIF. When delivered as LIF-NP, 200pg LIF opposed the effect of 20,000pg soluble IL-6, preventing *RORγt* induction. Indeed, a recent mathematical model estimated that the potency of a drug or cytokine could be increased by several orders of magnitude when the cytokine is delivered via a biodegradable particle targeted to T cells [23]

The next relevant question was, do the LIF-NP work *in vivo?* The answer is yes. Firstly, CD4-targeted LIF-NP biased the *in vivo* immune response towards tolerance, as measured in transgenic GFP-FOXP3 mice. The relative numbers of antigen-specific FOXP3+CD4+ T cells increased following the donor-specific transfusion (DST) of LIF-NP-coated cells targeted to CD4. This effect was in marked contrast to the use of IL-6-nano-treated lymphocytes in the DST system, where no increase of FOXP3+ cells occurred. Thus, the expansion of antigen-specific FOXP3+ cells was confirmed as being due to the specific bioactivity of LIF-nano and not a function of the delivery system itself. A more stringent test used mismatched vascularized heart allografts in mice. Here efficacy was also seen with LIF-NP treatment prolonging activity of the beating-heart graft.

Overall, the Park study [17] showed that the LIF / IL6 axis can be recapitulated in T cells treated with LIF-NP / IL6-NP, where the nanoparticulate therapeutic approach is underpinned by the targeted, controlled, sustained release of bioactive LIF, or IL-6, in low physiological doses within the precise microenvironment of the target cell. By targeting CD4+ T lymphocytes, the immune response of the fully immune competent mouse was manipulated. By extrapolation, selective targeting of other systems for specific biological responses would similarly produce target-specific effects – as is next presented for myelin repair where the target is the oligodendrocyte precursor cell that expresses neuroglial-2 (NG2) antigen and LIF-NP are targeted to NG2.

## 3. Multiple Sclerosis: A Case for Nanomedicine

Multiple sclerosis (MS) is a T cell-mediated inflammatory demyelinating disease of the human central nervous system (CNS). It is the most common disease of the CNS that affects young adults. Onset occurs between 20 – 40 years of age and the worldwide incidence is 2.5 million, with 2,500 cases being diagnosed each year in the UK alone. Scotland has the highest incidence of MS worldwide, with 1 in 170 women on the Orkney Islands being affected. Although the cause remains unknown, predisposing factors include being female and Caucasian, whilst the higher incidence in the northern and southern hemispheres suggests a link to vitamin D deficiency due to low sunlight, vitamin D being important in Treg-mediated self-tolerance [24–26]. For the patient, MS is a disease that, in its latter stages, is perceived to be worse than death.

### 3.1 MS is Caused by Both Autoimmunity and Demyelination of Nerve Axons

Treatment for MS is highly challenging. Not only must autoimmunity be reset to self-tolerance but also the structural damage to the demyelinated nerves must be repaired. The two are inextricably linked, since it is the immune-mediated demyelination of nerve axons that causes the neurological symptoms of MS. The loss of myelin's function for efficient electrical conductance along nerve axons, combined with the loss of myelin-derived metabolic support for the axon, eventually leads to the death of the nerve itself. In most cases, MS initially develops with alternating relapses and remissions of symptoms that reflect repeated waves of autoimmune neuro-inflammation. However, over time, nerve death increases, leading to irreversible progressive disease. In a few cases, the disease is immediately progressive. In the relapsing form, early remissions occur because the demyelination can be repaired endogenously (remyelination), restoring fast nerve axonal impulse conduction, together with protection and support of the axon. However, this myelin repair is inefficient and, over time, fails. Thus, although immunosuppressive therapies can reduce the probability of new relapses [27, 28], the progressive effects of demyelination remain untreatable.

**Table 1.** Multiple Sclerosis: evidence for a LIF/IL-6 axis in MS pathogenesis

EVIDENCE FOR TH17 / IL17 / IL-6 ACTIVITY IN MS	
(i)	The immune and neurotoxic effects of IL17 towards NG2+ glia provide crucial links the inflammatory and neurodegenerative aspects of MS.
	KANG ET AL 2013 [31]
(ii)	Infiltrating IL-17+ T cells are associated with active human disease.
	TZARTOS ET AL 2008 [45]
(iii)	*In vitro* treatment of CD4+ T cells from MS patients with LIF boosts Treg
	JANSSENS ET AL 2014 [46]
(iv)	BBB-endothelial cells (BBB-EC) IL-17 receptors in MS lesions; IL17 disrupts BBB tight junctions; TH17 cells transmigrate across BBB-ECs and promote CNS inflammation through CD4+ lymphocyte recruitment.
	KEBIR ET AL 2007 [47]
(v)	EAE model: increased Treg : TH17 ratio correlates with recovery of acute EAE.
	ALMONDA ET AL 2011 [48]
(vi)	EAE model: soluble LIF opposes TH17 immunity, reduces disease severity.
	CAO ET AL 2012 [33]
(vii)	IL-6:
	• TH17 immunity requires IL-6. BETTELLI ET AL 2010 [49] • EAE requires IL-6 but IL-6-null mouse that is resistant to EAE becomes sensitive to EAE if supplemented with exogenous IL-6. OKUDA ET AL 1999 [50] • EAE: site-specific IL-6 focuses inflammatory immunity in CNS QUINTANA ET AL 2009 [51]
MYELINATION	
(i)	EAE model: CNS-targeted LIF limits autoimmune-mediated demyelination.
	SLAETS ET AL 2010 [53]
(ii)	Demyelination model: delivery of LIF-viral increases reparative remyelination
	DEVERMAN AND PATTERSON 2012 [52]
(iii)	Demyelination model: delivery of LIF-NP increases reparative remyelination.
	RITTCHEN ET AL [36]

**Figure 3.**
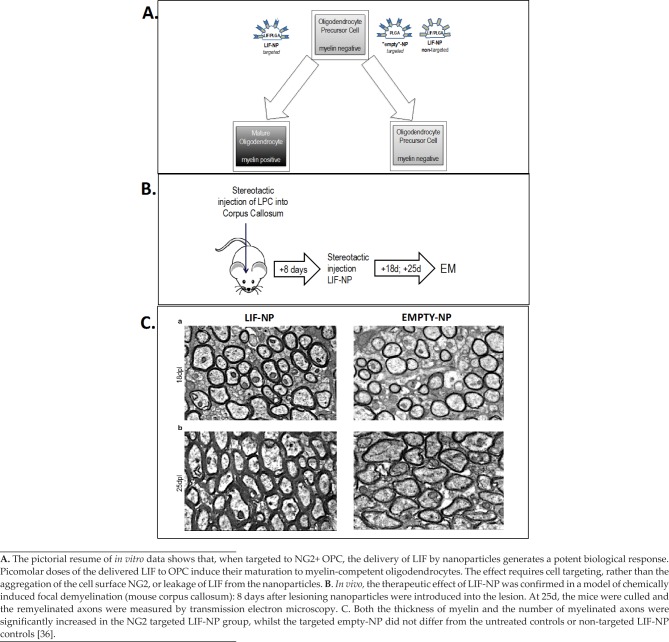
LIF-NP Promote Myelin Repair

### 3.2 Can the Endogenous Pathway for Myelin Repair Be Harnessed to Rescue Neurons? A Role for LIF

Since the removal of myelin renders axons metabolically non-viable, there is an intense need to understand the mechanisms by which myelin repair either occurs or fails. If LIF proves reparative, then therapy that uses LIF-NP becomes of prime importance. To date, we know that the natural repair pathway for remyelination of demyelinated axons involves endogenous oligodendrocyte precursor cells (OPCs). OPCs respond to demyelination by proliferation and migration to the area of damage; here they then differentiate into mature oligodendrocytes (OD) able to repair myelin. However, in MS, this repair process fails [29, 30]. This is partly due to becoming exhausted by the repeated attacks of demyelination and partly because of a critical block in the progression of OPCs to fully differentiated mature OD [31]. Can this maturation block be overcome? Accruing evidence indicates a central role for LIF.

LIF naturally occurs in the CNS and is implicated in both primary myelination and remyelination. LIF is released by astrocytes during activity-dependent cross-talk between axons OPCs and OD, during normal myelination [32]. It is known that treatment of inflammatory demyelination in experimental allergic encephalomyelitis (EAE, a model of MS) with soluble recombinant LIF or lentiviral overexpression of LIF in the CNS improves clinical outcome and decreases pathology. Moreover and unexpectedly, LIF is the underlying mechanism by which transplanted neural precursor cellS (NPC) reduce EAE symptoms [33]. It was shown that NPC-derived LIF opposed the differentiation of pathogenic TH17 cells *in vivo* – indeed, NPC cells can be replaced by recombinant LIF alone.

**Figure 4.**
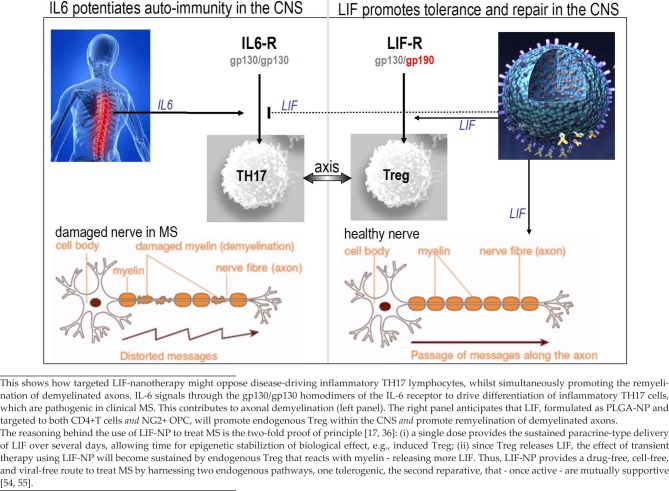
A pictorial summary of the LIF/IL-6 axis as it relates to MS

In marked contrast to LIF, interleukin-17 (IL17) – the signature cytokine of inflammatory TH17 cells that are present in MS lesions – has adverse effects on myelination [31]. Not only is IL-17 strongly inhibitory for the maturation of neuroglial-2 (NG2)-positive OPC to myelin synthesizing OD, but IL17 also reduces OPC survival. Thus, a single cytokine – IL-17 – provides a direct link between neuroinflammation and neurodegeneration in MS. This brings us back to the LIF (Treg) / IL-6 (TH17) axis and LIF's ability to oppose inflammatory TH17 cells. If there is a LIF / IL-6 axis in MS – as suggested by the evidence listed in Table 1 – then LIF's role in promoting reparative remyelination provides a route to treat both autoimmunity *and* demyelination in MS.

### 3.3 A Place for LIF-Nanotherapy As a Treatment Option for MS

A wide range of immunosuppressive treatment options are approved for the various types and stages of MS. These reduce the autoimmune attack and current disease modifyng therapies include beta-interferons, copaxone, fingolimid, nataluzimab and BG-12 (dimethyl fumarate). A recent addition is the monoclonal anti-CD52 antibody, alemtuzemab (previously known as CAMPATH-1H). However, all of these treatments have unwanted side effects. For alemtuzumab, secondary autoimmune disease is common. Alemtuzumab is a lymphodepleting antibody that spares bone marrow stem cells. In MS, it is used as part of a regimen of an induction therapy that first aims to deplete the entire immune system of the patient and, thereafter, allow the immune system to re-boot self-tolerance during homeostatic recovery. Initially, the patient is at high risk of infection. Thereafter, recovery is associated with significantly reduced disease. However, in a third of patients, secondary autoimmunities develop, most commonly of the thyroid. These appear to be driven by TH17 activity [34, 35], where IL-21 is produced by TH17 cells and drives IL-17 production. Since LIF opposes TH17 immunity, the concept of dual therapy – i.e., treating with alemtuzumab, followed by LIF-NP during homeostatic recovery phase – may enhance self-tolerant immunity and avoid secondary autoimmunity as the recovering T cell repertoire matures.

But, a far more important role for LIF-NP in MS therapy is myelin repair, which is currently untreatable. Rittchen et al. [36] showed that, *in vivo*, LIF-NP targeted to OPC increased the quality of myelin repair by both increasing numbers of remyelinated axons and increasing thickness of remyelinated axons. This proof of principle revealed two key points: (i) LIF that is delivered in the form of PLGA nanoparticles is bioactive within the brain (Figure 3) and (ii) a remarkably low concentration – picomolar range – of LIF induces a therapeutic remyelination effect. The *in vivo* remyelination study design was simplified by delivering LIF-NP directly into the demyelinated lesion of the corpus callosum. The next step is to establish a route for delivery of the targeted LIF-NP suitable for patients and that crosses the blood-brain-barrier (BBB). Given that the PLGA platform used for LIF-NP is the same as that used by BIND therapeutics (already approved by the FDA and in clinical trial to deliver cytotoxic agents), safety is unlikely to be an issue. This is especially the case, given the low dose of LIF and LIF's global reparative role at physiological levels. Considering the BBB, although this is defective in active MS lesions, sites that have become quiescent may be accessed by intranasal delivery. This is an optional delivery route that crosses the BBB at the cribiform plate at the back of the nasal cavity [37] and has been successful for PLGA nanoparticles (Figure 2). Alternatively, incorporation of the simil-opiod “7g” into the nanoparticles has been shown to promote uptake into the CNS via the opioid receptor following systemic delivery [38].

As a treatment for MS, LIF has three values: (i) opposition of TH17 immunity, (ii) repair of demyelinated axons and (iii) promotion of self-sustaining self-tolerance to myelin (Figure 4). By treating both autoimmunity and demyelination, LIF-NP provides a drug-free, cell-free, viral-free option to current strategies of treating MS. It is worth noting that, for myelin repair, there are two candidate therapeutics that are under trial: the repurposed antihistamine, clemastine fumarate [39], and a monoclonal antibody against Lingo-1 [40]. Neither of these provide the synergistic values of LIF-NP therapy.

## 4. Clinical Perspective

When considering LIF-nanotherapy in terms of translation the clinic, the two immediate considerations are (i) route of delivery and (ii) de-risking. Firstly, route of delivery of LIF-NP is linked to the therapeutic aim. For modulating the immune response in autoimmune disease, systemic delivery to circulating lymphocytes via the intravenous route is one option. Alternatively, direct delivery to specific sites – for example, the inflamed joint – may be selected. For skin conditions, such as psoriasis, a topical formulation would be appropriate. For treatment within the CNS, the challenge of overcoming the BBB becomes less of an issue with the success of intra-nasal delivery. Here, nanoparticle-formulated cytokines and growth factors are highly relevant to the treatment of both neurodegenerative and demyelinating diseases, with advantages over the cell-based therapeutic approach. Nanomedicine delivering biologics is imminent, following on the heels of nano-based platforms for delivery of drugs that are currently in clinical trials.

Secondly, in addition to standard pharmaceutical de-risking, safety is the central consideration for nanomedicine. As novel nanotherapeutic devices are developed, biocompatibility is crucial and biodegradation is a major advantage. This links to endogenous systems that can be harnessed by transient therapy, avoiding the potential of long-term physiological distortion due to the therapeutic (e.g., as may occur with viral-mediated delivery). A combination of nanotechnology with biological systems *in vivo* requires an understanding of the “deep” biology of the target and how this might be impacted by the candidate nanotherapeutic in holistic terms. The need for unifying expertise across the disciplines in order to maximize therapeutic gain, whilst avoiding any potential risk, is paramount. Such specialized de-risking will run alongside established de-risking strategies for a novel biologic therapeutic. For PLGA-based technology, approval by the FDA and the current clinical trials being run by BIND Therapeutics are encouraging.

## 5. Conclusion

The therapeutic potential of LIF enabled by formulation as PLGA nanoparticles extends to tolerogenesis [17]; support of cell and tissue grafts [41–43]; and repair of myelin [36]. Untested is the ability of LIF-NP to synergise with other nanotherapeutics, though LIF-NP when combined with XAV939-NP (a wnt signalling inhibitor) revealed that neural precursor cell lineages are sensitive to manipulation [44]. Although arguably premature as an overview, the progress of LIF-NP in nanomedicine is well underway and future major indications include the neurodegenerative diseases where LIF may reduce inflammatory pathogenesis and be prosurvival for neurons, for example in Parkinson's and Alzheimer's Diseases; also the retinopathies, where LIF's neurogenic and neuroprotective properties may reduce loss of vision by supporting the retinal pigment epithelium of the eye.

Finally, the question arises – can LIF-NP act on an endogenous stem and precursor cells for tissue repair? This would provide a simple defined route to reduce or even replace the need for exogenous delivery of therapeutic stem cells in regenerative medicine.

## 6. Dedication

We dedicate this overview to Paul Patterson (1944–2014), who first recognized LIF's role in neuro-immunity and, more recently, identified maternal IL-6 as being linked to the risk of autism and schizophrenia in offspring, two amongst many of Paul's groundbring discoveries now being pursued worldwide.
